# Periodontal Outcomes in Orthodontic Patients With Skeletal Malocclusions Undergoing Orthognathic Surgery: A Comprehensive Review

**DOI:** 10.7759/cureus.111108

**Published:** 2026-06-18

**Authors:** Salem Alluhaidan, Husam Tawfeeq, Joud Alrajhi, Nouf Qaisi, Abdulaziz Alshanqiti, Abdulrahman Al Saadoun, Faris Alharbi, Hamad Alajmi, Abdolkareem Almobayed, Abdulaziz Alolayan, Anas Alquraishi, Suha Alsharyoufi, Rawan S. Alrehaili, Almothana Almulhim, Alaa Alauti

**Affiliations:** 1 College of Dentistry, Imam Abdulrahman Bin Faisal University, Dammam, SAU; 2 College of Dentistry, King Saud bin Abdulaziz University for Health Sciences, Riyadh, SAU; 3 Faculty of Dentistry, King Abdulaziz University, Jeddah, SAU; 4 Family Dentistry, Private Practice, Medina, SAU; 5 College of Dentistry, King Faisal University, Al-Ahsa, SAU; 6 Orthodontics, Maleen Consultant Center, Riyadh, SAU

**Keywords:** gingival recession, orthodontic decompensation, orthognathic surgery, periodontal outcomes, presurgical orthodontics, skeletal malocclusion

## Abstract

Periodontal outcomes in orthodontic patients undergoing orthognathic surgery reflect a biologically complex treatment pathway rather than a single treatment-related event. This structured, comprehensive narrative review examined the available evidence on periodontal health in patients with skeletal malocclusions treated with combined orthodontic-orthognathic therapy, with emphasis on baseline periodontal vulnerability, presurgical orthodontic decompensation, surgery-related tissue response, post-treatment periodontal stability, and the major factors influencing prognosis. The search was conducted from database inception through May 2026. Evidence from 35 studies identified through PubMed, Scopus, Web of Science, and Embase was reviewed and interpreted comparatively according to treatment phase, periodontal outcome type, anatomic site, and level of clinical support. The evidence showed that periodontal risk was not distributed evenly across the treatment sequence. Baseline vulnerability was frequently present before treatment, particularly in the mandibular anterior region, where reduced alveolar support, thin phenotype, and unfavorable root position were common. Presurgical orthodontic decompensation emerged as the phase most consistently associated with periodontal challenge, especially in relation to alveolar bone loss, dehiscence, and fenestration around mandibular incisors. By contrast, orthognathic surgery itself appeared to contribute more often to short-term plaque-related and soft-tissue changes than to generalized long-term periodontal breakdown, although segmental procedures may carry greater site-specific risk. Overall, the current literature supports a model in which periodontal prognosis is phase-dependent, anatomically conditioned, and clinically modifiable. The strongest evidence points to the importance of early periodontal risk assessment, biologically controlled decompensation, and close interdisciplinary monitoring throughout orthodontic-surgical treatment.

## Introduction and background

Combined orthodontic-orthognathic treatment remains the standard approach for patients with dentofacial discrepancies that exceed the biologic and esthetic limits of orthodontic camouflage and require coordinated correction of the skeletal bases and dental arches [1]. In the conventional pathway, presurgical orthodontics is not merely preparatory but biologically decisive, because it reveals the underlying skeletal discrepancy by removing dentoalveolar compensation and thereby determines the conditions under which surgery will be performed [2]. For that reason, the quality of the final orthodontic-surgical result depends on both surgical accuracy and whether decompensation and alignment are carried out within a biologically defensible dentoalveolar envelope [3]. Within this framework, the periodontal dimension has received less focused attention than skeletal, occlusal, and facial outcomes, despite the fact that orthognathic patients are typically treated in early adulthood, when periodontal phenotype, mucogingival stability, and alveolar limitations become more relevant to the tolerance of tooth movement [4].

Current periodontal evidence supports that phenotype is a biologic modifier that may influence the likelihood of recession, attachment loss, and soft-tissue instability when orthodontic movement is directed toward the cortical boundaries [5]. This issue becomes important in orthognathic treatment because presurgical decompensation intentionally moves teeth away from their compensated positions and may therefore challenge sites that are already anatomically limited before surgery begins [6]. The broader orthodontic literature suggests that periodontal consequences may not be fully apparent at the end of active treatment. In a long-term follow-up study, Renkema et al. reported that labial gingival recession increased from 7% at the end of orthodontic treatment to 20% after two years and 38% after five years, indicating that periodontal change may evolve beyond the active appliance phase [7]. At the same time, recent systematic and consensus-level evidence indicates that orthodontic treatment can be performed successfully in periodontally vulnerable patients when inflammation is controlled, biomechanics are adapted, and interdisciplinary periodontal management is integrated into treatment planning [8,9]. This distinction is clinically important for orthognathic patients, because the relevant question is not simply whether periodontal risk exists, but rather where that risk is concentrated and which patients are least able to tolerate it.

Emerging evidence on orthodontic-associated alveolar defects further suggests that adult orthodontic patients may present with pre-existing dehiscence or fenestration, or develop such defects during treatment when root movement exceeds the available alveolar housing [6,10]. That concept is particularly relevant to orthognathic patients, in whom decompensation is often more extensive and more anatomically demanding than in routine nonsurgical treatment, especially in skeletal Class II and Class III patterns with pre-existing dentoalveolar compensation [11,12]. However, the orthognathic-periodontal literature remains fragmented. Some studies focus on gingival recession, probing depth, plaque accumulation, or bleeding indices, whereas others emphasize cone-beam computed tomography (CBCT)-derived measurements of alveolar thickness, alveolar height, dehiscence, or fenestration, making direct synthesis difficult [13]. More importantly, relatively few studies separate the periodontal effects of baseline anatomy, presurgical orthodontics, surgery itself, and postsurgical finishing with sufficient clarity to determine where the principal biologic burden actually lies [13,14]. Recent orthognathic-specific evidence also suggests that periodontal risk may be localized rather than generalized, with the mandibular anterior region emerging as a particularly susceptible site in skeletal Class III patients, especially when periodontal phenotype is thin and alveolar dimensions are limited [14]. Accordingly, a focused review is warranted. The present comprehensive narrative review examines periodontal outcomes in orthodontic patients with skeletal malocclusions undergoing orthognathic surgery, with emphasis on baseline periodontal vulnerability, periodontal risk during orthodontic decompensation, surgery-related tissue response, post-treatment periodontal stability, and the clinical factors that appear to modify prognosis.

## Review

Review design and scope

This article was developed as a structured, comprehensive narrative review focused on periodontal outcomes in orthodontic patients with skeletal malocclusions undergoing orthognathic surgery. The review was designed to examine periodontal health as a dynamic outcome influenced by the full orthodontic-surgical pathway, including pretreatment anatomy, presurgical orthodontic decompensation, surgery-related tissue response, and post-treatment stability. The central aim was to synthesize the literature across the major phases and determinants of periodontal risk in this population, while distinguishing clinically meaningful periodontal outcomes from radiographic or surrogate findings that may not directly reflect long-term periodontal breakdown. The review was restricted to periodontal questions arising within combined orthodontic-orthognathic care. General surgical outcomes, skeletal correction, airway change, esthetic outcomes, and occlusal endpoints were considered only when they had a direct periodontal implication. The review was therefore organized around the principal domains that emerged as most relevant to the topic: baseline periodontal vulnerability in skeletal malocclusion patients, periodontal risk during orthodontic decompensation, periodontal tissue response to orthognathic surgery, post-treatment periodontal outcomes over time, modifying factors influencing prognosis, interdisciplinary clinical implications, and the major limitations of the current evidence base.

Information sources and literature search

A targeted search of the literature was undertaken in major biomedical databases, including PubMed, Scopus, Web of Science, and Embase, with additional manual screening of the reference lists of relevant reviews and key primary studies. The initial strategy was developed in PubMed/MEDLINE and then adapted for Scopus, Web of Science Core Collection, and Embase using the corresponding indexing terms and field tags of each database (Table 1).

**Table 1:** Database-specific electronic search strategies

Database	Search strategy
PubMed	("Orthodontics"[Mesh] OR orthodontic treatment OR presurgical orthodontic* OR preoperative orthodontic* OR orthodontic decompensation OR decompensation OR postsurgical orthodontic* OR orthodontic-surgical treatment OR combined orthodontic-orthognathic treatment) AND ("Orthognathic Surgery"[Mesh] OR orthognathic surg* OR orthognathic surgical procedure* OR "Le Fort I" OR maxillary osteotomy OR mandibular osteotomy OR "bilateral sagittal split osteotomy" OR BSSO OR sagittal split ramus osteotomy OR genioplasty OR segmental osteotomy OR anterior segmental osteotomy OR surgery-first) AND (skeletal malocclusion* OR dentofacial deformit* OR jaw deformit* OR "Malocclusion, Angle Class II"[Mesh] OR "Malocclusion, Angle Class III"[Mesh] OR skeletal class II OR skeletal class III OR mandibular prognathism OR mandibular retrognathism OR maxillary deficiency OR vertical maxillary excess OR open bite) AND ("Periodontium"[Mesh] OR periodontal OR periodontium OR gingiv* OR gingival recession OR mucogingiv* OR periodontal phenotype OR gingival phenotype OR alveolar bone OR alveolar housing OR alveolar bone loss OR dehiscence OR fenestration OR clinical attachment loss OR probing depth OR bleeding on probing OR plaque index OR keratinized tissue OR attached gingiva)
Scopus	TITLE-ABS-KEY ( orthodontic treatment OR presurgical orthodontic* OR preoperative orthodontic* OR orthodontic decompensation OR decompensation OR postsurgical orthodontic* OR orthodontic-surgical treatment OR combined orthodontic-orthognathic treatment ) AND TITLE-ABS-KEY ( orthognathic surg* OR orthognathic surgical procedure* OR "Le Fort I" OR maxillary osteotomy OR mandibular osteotomy OR "bilateral sagittal split osteotomy" OR BSSO OR sagittal split ramus osteotomy OR genioplasty OR segmental osteotomy OR anterior segmental osteotomy OR surgery-first ) AND TITLE-ABS-KEY ( skeletal malocclusion* OR dentofacial deformit* OR jaw deformit* OR skeletal class II OR skeletal class III OR mandibular prognathism OR mandibular retrognathism OR maxillary deficiency OR vertical maxillary excess OR open bite ) AND TITLE-ABS-KEY ( periodontal OR periodontium OR gingiv* OR gingival recession OR mucogingiv* OR periodontal phenotype OR gingival phenotype OR alveolar bone OR alveolar housing OR alveolar bone loss OR dehiscence OR fenestration OR clinical attachment loss OR probing depth OR bleeding on probing OR plaque index OR keratinized tissue OR attached gingiva )
Web of Science Core Collection	TS=(orthodontic treatment OR presurgical orthodontic* OR preoperative orthodontic* OR orthodontic decompensation OR decompensation OR postsurgical orthodontic* OR orthodontic-surgical treatment OR combined orthodontic-orthognathic treatment) AND TS=(orthognathic surg* OR orthognathic surgical procedure* OR "Le Fort I" OR maxillary osteotomy OR mandibular osteotomy OR "bilateral sagittal split osteotomy" OR BSSO OR sagittal split ramus osteotomy OR genioplasty OR segmental osteotomy OR anterior segmental osteotomy OR surgery-first) AND TS=(skeletal malocclusion* OR dentofacial deformit* OR jaw deformit* OR skeletal class II OR skeletal class III OR mandibular prognathism OR mandibular retrognathism OR maxillary deficiency OR vertical maxillary excess OR open bite) AND TS=(periodontal OR periodontium OR gingiv* OR gingival recession OR mucogingiv* OR periodontal phenotype OR gingival phenotype OR alveolar bone OR alveolar housing OR alveolar bone loss OR dehiscence OR fenestration OR clinical attachment loss OR probing depth OR bleeding on probing OR plaque index OR keratinized tissue OR attached gingiva)
Embase	('orthodontics'/exp OR orthodontic treatment:ti,ab,kw OR presurgical orthodontic*:ti,ab,kw OR preoperative orthodontic*:ti,ab,kw OR orthodontic decompensation:ti,ab,kw OR decompensation:ti,ab,kw OR postsurgical orthodontic*:ti,ab,kw OR orthodontic-surgical treatment:ti,ab,kw OR combined orthodontic-orthognathic treatment:ti,ab,kw) AND ('orthognathic surgery'/exp OR orthognathic surg*:ti,ab,kw OR orthognathic surgical procedure*:ti,ab,kw OR 'Le Fort I':ti,ab,kw OR maxillary osteotomy:ti,ab,kw OR mandibular osteotomy:ti,ab,kw OR 'bilateral sagittal split osteotomy':ti,ab,kw OR BSSO:ti,ab,kw OR sagittal split ramus osteotomy:ti,ab,kw OR genioplasty:ti,ab,kw OR segmental osteotomy:ti,ab,kw OR anterior segmental osteotomy:ti,ab,kw OR surgery-first:ti,ab,kw) AND ('skeletal malocclusion':ti,ab,kw OR dentofacial deformit*:ti,ab,kw OR jaw deformit*:ti,ab,kw OR 'angle class ii malocclusion'/exp OR 'angle class iii malocclusion'/exp OR skeletal class II:ti,ab,kw OR skeletal class III:ti,ab,kw OR mandibular prognathism:ti,ab,kw OR mandibular retrognathism:ti,ab,kw OR maxillary deficiency:ti,ab,kw OR vertical maxillary excess:ti,ab,kw OR open bite:ti,ab,kw) AND ('periodontium'/exp OR periodontal:ti,ab,kw OR periodontium:ti,ab,kw OR gingiv*:ti,ab,kw OR gingival recession:ti,ab,kw OR mucogingiv*:ti,ab,kw OR periodontal phenotype:ti,ab,kw OR gingival phenotype:ti,ab,kw OR alveolar bone:ti,ab,kw OR alveolar housing:ti,ab,kw OR alveolar bone loss:ti,ab,kw OR dehiscence:ti,ab,kw OR fenestration:ti,ab,kw OR clinical attachment loss:ti,ab,kw OR probing depth:ti,ab,kw OR bleeding on probing:ti,ab,kw OR plaque index:ti,ab,kw OR keratinized tissue:ti,ab,kw OR attached gingiva:ti,ab,kw)

Eligibility and source selection

Sources were considered eligible when they addressed periodontal status, periodontal change, alveolar support, or periodontal risk factors in patients with skeletal malocclusions treated with orthodontics in conjunction with orthognathic surgery. Eligible articles included prospective and retrospective clinical studies, cohort studies, comparative studies, imaging-based investigations, and systematic reviews relevant to the review question. Because a substantial part of the literature in this field relies on dentoalveolar imaging rather than conventional periodontal endpoints, studies evaluating alveolar bone thickness, alveolar bone height, dehiscence, fenestration, or root position relative to the cortical envelope were also included when they were clearly related to the orthodontic-surgical context and contributed meaningfully to the understanding of periodontal vulnerability. Studies were excluded if they focused exclusively on non-surgical orthodontic populations without relevance to skeletal surgical treatment, reported only general orthognathic outcomes without periodontal interpretation, or addressed periodontal disease in a way unrelated to orthodontic-surgical management.

Data interpretation and synthesis

The synthesis was narrative and organized into thematic domains. Evidence was interpreted comparatively across the major clinical stages of treatment and according to the type of periodontal outcome reported. Within each thematic domain, the literature was synthesized in relation to the periodontal site involved, the phase of treatment, type of skeletal discrepancy, direction of tooth movement, and major modifying factors such as vertical pattern, periodontal phenotype, oral hygiene, and treatment mechanics.

Baseline periodontal vulnerability in skeletal malocclusion patients

Baseline periodontal vulnerability in orthognathic patients should not be viewed as a secondary issue that appears only after orthodontic decompensation begins. Kook et al. compared 20 normal-occlusion subjects with 20 skeletal Class III surgical patients and found significantly greater vertical alveolar bone loss in the Class III group [15]. They also showed that mandibular incisors had more alveolar bone loss than maxillary incisors in both groups, with the difference being especially pronounced on the lingual side in the Class III patients. At the apical level, alveolar bone thickness was generally greater than cemento-enamel junction (CEJ) width, except for the mandibular incisors in the skeletal Class III group, where the percentage fell to 81.33%. These findings suggest that the mandibular anterior segment in Class III surgical patients may begin treatment with less hard-tissue reserve than is often assumed. Kim et al. reported a similar baseline pattern in 20 skeletal Class III patients evaluated before orthognathic surgery [16]. In their study, mandibular incisors showed reduced vertical alveolar bone levels compared with maxillary incisors, again with greater compromise on the lingual side. The percentage of vertical bone loss relative to root length was significantly higher in the lower incisors than in the upper incisors. Oh et al. strengthened this point in untreated skeletal Class III patients [17]. They reported that labial alveolar bone loss was 34.4% in the mandible versus 21.8% in the maxilla, whereas lingual bone loss was 27.6% versus 18.3%, respectively. They also found that labial alveolar bone thickness was less than 1.0 mm at the level corresponding to 70% of root length for all incisors. This suggests that baseline vulnerability is not confined to isolated defects. In some patients, it reflects a generally thin anterior alveolar housing.

Yagci et al. expanded the discussion beyond Class III malocclusion by examining 3444 teeth across skeletal Class I, II, and III groups [18]. They found no significant intergroup difference in overall dehiscence prevalence, but they did report more mandibular alveolar defects in Class II and Class III subjects, at 41.11% and 45.02%, respectively. They also showed that dehiscences occurred most frequently in the mandibular incisors across all groups. At the same time, this study showed that fenestration behaved differently from dehiscence. Fenestration was more prevalent in the maxilla, and the Class II group had a significantly greater prevalence of fenestration than the other groups. This distinction matters because not all alveolar defects follow the same anatomical pattern. Dehiscence appears to cluster more in the mandible, whereas fenestration may follow a different distribution. Vertical facial pattern seems to modify this baseline susceptibility. Han et al. evaluated 84 skeletal Class III patients, including 28 hypodivergent, 28 normodivergent, and 28 hyperdivergent subjects [19]. They found that both dehiscence and fenestration were more prevalent in the hyperdivergent group than in the hypo- or normodivergent groups. Their results suggest that sagittal discrepancy alone does not explain periodontal vulnerability. Vertical growth pattern appears to intensify it. Soft-tissue phenotype adds another dimension that bone-focused studies cannot fully capture. Jing et al. found that the prevalence of a thin periodontal biotype in skeletal Class III patients was 33.9% in the anterior region, and the mean width of keratinized gingiva was 4.37 mm [20]. They also showed that biotype was significantly associated with dental arch and the width of keratinized gingiva. This is clinically relevant because a limited hard-tissue envelope and a thin soft-tissue phenotype may coexist in the same patient. The CBCT-based baseline literature is informative, but it should not be overinterpreted. Patcas et al. showed that although CBCT measurements were generally close to physical measurements, the limits of agreement could still reach 2.10 mm, and even 0.125-mm voxel CBCT did not depict very thin buccal bone reliably [21]. They specifically warned that CBCT can overestimate dehiscences and fenestrations. Alsino et al. later confirmed this concern in a surgical-exposure validation study [22]. They found that CBCT diagnosed dehiscence in 42.7% of sites versus 17.7% on direct examination, and fenestration in 39.5% versus 13.5%, respectively. Clinically, this is a critical warning: CBCT identified dehiscence at approximately 2.5 times the direct surgical-exposure rate and fenestration at almost three times the direct rate. Therefore, CBCT-detected defects should not automatically be interpreted as clinically established periodontal breakdown. A radiographic dehiscence or fenestration, particularly in very thin cortical bone, may represent an imaging overcall or an anatomic vulnerability rather than active disease. This distinction is important because overinterpretation of CBCT findings may lead clinicians to overestimate periodontal risk, overtreat radiographic defects, or modify orthodontic-surgical plans unnecessarily in the absence of gingival recession, clinical attachment loss, inflammation, or progressive marginal tissue change. This limitation changes how the baseline literature should be synthesized.

The existing studies support the presence of a restricted alveolar envelope in many skeletal malocclusion patients, especially around the mandibular incisors. However, they do not prove that all radiographic defects represent clinically manifest periodontal disease. The strongest conclusion at this stage is therefore anatomical rather than disease-based: many orthognathic patients begin treatment with less periodontal reserve than ideal, and this reserve is particularly limited in the mandibular anterior region. Overall, three points appear most defensible. First, baseline periodontal vulnerability is real and is often present before treatment begins. Second, the mandibular incisor region is the most consistently vulnerable site across the available studies. Third, this vulnerability seems to be amplified by hyperdivergence, thin periodontal phenotype, and unfavorable root position within the alveolar housing. What remains uncertain is how strongly these baseline anatomic findings predict later gingival recession, attachment loss, or long-term periodontal instability during combined orthodontic-surgical treatment.

Periodontal changes during presurgical orthodontic decompensation

Presurgical orthodontic decompensation is the phase most likely to challenge the periodontal envelope in combined orthodontic-surgical treatment. Unlike routine alignment, it often requires reversal of long-standing dentoalveolar compensation so that the teeth can be repositioned over their skeletal bases before surgery. In practice, this means that incisors are frequently moved toward already thin cortical boundaries, particularly in the mandibular anterior region. He et al., in a systematic review and meta-analysis, concluded that presurgical orthodontic decompensation in skeletal Class III patients was associated with significant alveolar bone loss in mandibular incisors on both the lingual and labial sides, with more pronounced loss on the lingual side at the apex level and on the labial side near the crown [23]. They also found no significant bone loss during postsurgical orthodontic treatment. Lee et al. provided some of the clearest phase-specific data [24]. In 25 adults with mandibular prognathism, they assessed the lower incisors at pretreatment, after presurgical orthodontics, and after debonding. The mean lingual vertical bone level of the lower central incisor increased from 1.38±0.61 mm at baseline to 2.32±1.07 mm after presurgical orthodontics, then improved slightly to 1.91±0.95 mm after treatment. For the lower lateral incisor, the corresponding lingual values were 1.47±0.81 mm, 2.45±1.60 mm, and 1.67±0.72 mm. The same study showed that labial midroot bone thickness of the lower lateral incisor fell from 0.70±0.19 mm to 0.42±0.24 mm during decompensation, with only partial recovery to 0.54±0.19 mm later. Lingual apical thickness of that tooth also decreased from 3.88±1.28 mm to 2.67±0.98 mm and remained at 2.63±1.11 mm at the end of treatment. The study therefore suggested that the major periodontal cost was concentrated in the presurgical phase, whereas the postsurgical phase did not produce further significant deterioration and yielded only limited recovery. Sun et al. reached a closely related conclusion in adults with skeletal Class III malocclusion [25]. They reported that after presurgical orthodontic decompensation, alveolar bone thickness at the apex became thinner and vertical alveolar height was reduced, particularly on the labial side. They also linked these changes to lower-incisor inclination, reinforcing the idea that decompensation can alter the surrounding bone architecture even before surgery occurs. Yao et al. added to this by showing that the periodontal effect of decompensation is not determined by sagittal movement alone [26].

In 29 Class III patients awaiting two-jaw surgery with an average presurgical treatment time of 11 months, they found that alveolar bone height generally decreased after decompensation regardless of mandibular plane angle. However, the vertical facial pattern modified the amount and location of the change. In their multivariate model, buccal thickness change was 0.734 mm lower in the average-angle group and 0.947 mm lower in the high-angle group than in the low-angle group. At the same time, each 1° increase in proclination was associated with a 0.097 mm increase in buccal thickness change but a 0.137 mm decrease in lingual thickness change. Demirsoy et al. further challenged the idea that periodontal risk can be predicted from inclination change alone [27]. In 26 adults with skeletal Class III deformity, they examined 208 incisors after presurgical orthodontics and found dehiscence, fenestration, or both in 81 upper incisors and 94 lower incisors, whereas only 23 upper and 10 lower incisors were free of such defects. However, they did not find a significant linear relationship between the amount of inclination change and defect formation. This helps explain why studies that look only at cephalometric proclination or retroclination often fail to capture the full biologic picture. The same concern is not limited to skeletal Class III cases. Fu et al. examined 30 high-angle skeletal Class II patients treated with preoperative orthodontics and orthognathic surgery and concluded that ideal mandibular anterior decompensation could be achieved, but that lingual periodontal risk increased during that process [28]. This broadens the interpretation of the literature. The problem is not unique to Class III mandibular incisor proclination. Rather, whenever decompensation moves incisors toward a cortical boundary, the vulnerable surface depends on the original compensation pattern and the direction of correction. A further point of synthesis is that the evidence for decompensation-related risk is stronger for hard-tissue imaging outcomes than for clinically important soft-tissue breakdown.

He et al. concluded that gingival recession after presurgical decompensation was generally clinically acceptable, even though alveolar bone loss was significant on imaging [23]. It is worth mentioning that radiographic thinning or increased dehiscence does not automatically mean that the patient will develop progressive recession or attachment loss. At present, the literature supports periodontal vulnerability during decompensation more convincingly than it supports inevitable clinical periodontal damage. Ahn et al. reported in 15 skeletal Class III patients treated with augmented corticotomy before surgery that mandibular incisors were proclined by 10.45°, the incisal edge moved labially by 3.47 mm, and alveolar bone thickness increased by 1.56 mm at the root apex and 1.98 mm at the B-point level, without gingival recession [29]. Ma et al. later compared traditional presurgical orthodontics with augmented corticotomy in 50 high-angle skeletal Class III patients [30]. Baseline fenestration and dehiscence rates were 39.24% and 24.10%, respectively. After presurgical orthodontic treatment, fenestration rose to 49.83% in the traditional group but remained 25.86% in the augmented corticotomy group, while dehiscence reached 58.08% versus 32.07%, respectively. These studies do not justify routine augmentation for every patient, but they do show that periodontal deterioration during decompensation is not necessarily unavoidable. Most orthognathic-periodontal studies focus on sagittal decompensation of the incisors, especially the mandibular incisors. By contrast, direct evidence on transverse coordination, arch expansion, and broader arch-form correction in orthognathic cohorts is much thinner. For that reason, the strongest and most defensible conclusion at present is a narrower one: presurgical decompensation can compromise alveolar support, particularly around mandibular incisors and particularly in patients with a thin envelope, but the magnitude and clinical expression of that risk depend on more than the amount of tooth inclination change alone.

Periodontal responses associated with orthognathic surgery

The periodontal effect of orthognathic surgery is more difficult to isolate than the effect of presurgical decompensation. By the time surgery is performed, the teeth and supporting tissues have already been exposed to months of orthodontic movement. As a result, a postoperative periodontal finding may reflect the surgery itself, the preceding decompensation, short-term hygiene deterioration, or a combination of these factors. This helps explain why the literature on surgery-specific periodontal injury remains less definitive than the literature on decompensation-related alveolar change. Mester et al., in a systematic review that included only seven observational clinical studies with sample sizes ranging from 24 to 40 patients, concluded that gingival recession outcomes after combined orthodontic-orthognathic treatment were inconsistent and that the overall certainty of evidence was low to moderate [13]. The most consistent surgery-related finding is a short-term deterioration in periodontal conditions during the early postoperative phase. Weinspach et al. followed 15 consecutively treated patients with a mean age of 24.9±7.7 years who underwent Le Fort I osteotomy, sagittal split osteotomy, or both [31]. Plaque index increased during the first postoperative week and then decreased by six weeks. Oral-site probing depth also increased during this interval. Buccal gingival recession rose from 0.10±0.16 mm at baseline to 0.21±0.23 mm after one week and to 0.31±0.31 mm after six weeks. By contrast, bleeding on probing did not change meaningfully. These findings suggest that the immediate postoperative period is associated mainly with transient plaque accumulation, soft-tissue alteration, and localized probing-depth change rather than with generalized periodontal destruction. Although Weinspach et al. documented a measurable increase in gingival recession, the magnitude remained small and was not considered clinically important from an esthetic perspective. The interpretation becomes more complex when segmental procedures are considered. In these cases, periodontal risk may become site-specific because interdental osteotomies can directly affect roots, interdental bone, and papillary tissues. Schultes et al. evaluated 30 Class II patients four to 10 years after segmental orthognathic surgery and identified 51 pathologic periodontal lesions at 74 segmental osteotomy sites [32]. These lesions included 35 osseous periodontal defects and 16 missing teeth. On this basis, they concluded that interdental osteotomies were associated with a high incidence of dental and periodontal trauma.

Later studies, however, suggest that this risk is not uniform and may depend heavily on technique and perioperative management. Ueki et al. studied 40 adults who underwent anterior segmental or dento-osseous osteotomy [33]. In the group managed with their specialized orthodontic-periodontal protocol, they reported no gingival-margin degeneration and no periodontal defects at the osteotomy site. They also found that alveolar bone height increased in the managed group but decreased in the conventionally treated group after maxillary osteotomy. These findings suggest that periodontal damage around segmental osteotomy sites is not necessarily an inevitable consequence of segmentation itself. Ho et al. reported comparable conclusions in a retrospective series of 85 consecutive patients treated with segmental Le Fort I osteotomy [34]. Their overall complication rate was 27%, but only three patients developed minor periodontal defects, three had devitalized teeth, and one experienced tooth loss. They found no overall segmental loss of bone or teeth and considered segmental maxillary surgery safe in carefully selected cases. When these results are considered together with those of Schultes et al., the literature suggests that periodontal outcomes after segmental surgery vary substantially across studies and are likely influenced by case selection, presurgical orthodontic root divergence, surgical design, root proximity, and perioperative management [32]. The high complication rate reported in older studies should therefore not be interpreted as an inherent or unavoidable effect of segmentation. With modern presurgical orthodontic coordination, improved interdental root separation, precision osteotomy techniques, surgical templates, and rigid internal fixation, the periodontal risk of segmental surgery is likely reduced. The key operator-dependent risk factor is root proximity at the interdental osteotomy site, because inadequate root separation may lead to root injury, interdental bone loss, papillary compromise, or localized periodontal defects.

Contemporary segmental surgery should therefore be considered technique-sensitive and risk-modifiable rather than uniformly periodontally destructive. Orthognathic surgery can produce short-term periodontal changes, particularly plaque accumulation, localized probing-depth increase, and small increases in gingival recession during early healing. However, the current evidence does not support viewing orthognathic surgery alone as a major independent cause of generalized periodontal breakdown. The main area of greater concern is segmental surgery, where periodontal effects may be more pronounced but also appear to be more technique-sensitive. The most appropriate interpretation is therefore that orthognathic surgery represents a temporary periodontal stress period rather than a uniformly destructive periodontal event. In nonsegmental cases, the dominant pattern is usually short-term hygiene-related and soft-tissue change. In segmental cases, the periodontal consequences may be more clinically relevant, but the available evidence suggests that they depend greatly on surgical execution and interdisciplinary management.

Periodontal outcomes after orthodontic treatment and during follow-up

Post-treatment periodontal outcomes should be interpreted in two distinct phases. The early postoperative phase is characterized mainly by transient plaque-related and soft-tissue changes, whereas the evidence for durable periodontal breakdown after completion of combined treatment is much less consistent. In other words, short-term deterioration is reported more regularly than long-term irreversible periodontal harm. This distinction is central to the literature and should not be blurred. De Paulo et al. concluded in their systematic review that gingival recession in the range of 0.5 mm to 3.0 mm was a common finding after combined orthodontic-orthognathic treatment, mainly in incisors, yet they also stated that true recession was not generally attributable to orthognathic surgery itself [35]. Mester et al. reached a similarly cautious conclusion and found that recession outcomes across seven observational studies remained inconsistent, with only low-to-moderate certainty of evidence [13]. The short-term pattern is clearer. Weinspach et al. followed 15 patients through the early postoperative period and found that plaque accumulation worsened during the first week after surgery, then improved by six weeks [31]. In the same cohort, buccal gingival recession increased from 0.10±0.16 mm at baseline to 0.21±0.23 mm after one week and to 0.31±0.31 mm after six weeks. Not all studies, however, show persistent worsening beyond the earliest healing phase.

Esen et al. evaluated 10 orthognathic surgery patients at baseline, three months, and six months after surgery [36]. Their cohort included six double-jaw procedures, two sagittal split ramus osteotomies, and two Le Fort I osteotomies. They found no significant difference over time in plaque index, gingival index, probing depth, bleeding on probing, clinical attachment level, or mobility at the maxillary, mandibular, or full-mouth level. This study is limited by its small sample, but it remains important because it suggests that early postoperative changes do not necessarily persist as measurable full-mouth periodontal deterioration at medium-term follow-up. The literature becomes more complex when outcomes are assessed at the end of treatment rather than only during early healing. De Leyva et al. randomized 28 patients undergoing orthognathic surgery to postsurgical treatment with either fixed appliances or clear aligners [37]. At the end of treatment, the aligner group showed better plaque, bleeding-on-probing, and probing-depth outcomes, while total treatment duration was similar between groups. This trial suggests that some post-treatment periodontal findings remain modifiable and are influenced by the nature of the finishing phase, particularly by how easily the patient can maintain hygiene. A plausible explanation is that clear aligners reduce the plaque-retentive burden of postsurgical fixed appliances, which may include brackets, ligatures, archwires, surgical hooks, complex elastics, and intermaxillary fixation auxiliaries. Because aligners are removable, they allow better access for oral hygiene during early tissue healing, when edema, discomfort, limited opening, and surgical splints may already compromise plaque control. This appliance-related advantage may partly explain the better plaque, bleeding-on-probing, and probing-depth outcomes reported with aligners, although attachments, buttons, cut-outs, or elastics may still be required in some cases. Longer-term evidence is still more heterogeneous than early postoperative evidence. Liu et al. studied 33 skeletal Class III patients and 131 mandibular incisors after orthodontic-orthognathic treatment [14]. In the group without periodontally accelerated osteogenic orthodontics (PAOO), gingival thickness decreased by 0.15±0.21 mm, and keratinized gingival width decreased by 0.74±0.91 mm. In the PAOO group, gingival thickness increased by 0.32±0.28 mm and keratinized gingival width increased by 2.09±1.51 mm. Most notably, the proportion of gingival recession increased by 47.62% in the non-PAOO group and was reported to be 14.77 times that of the PAOO group. The study also identified possible risk thresholds, including gingival thickness below 0.72 mm, alveolar bone height above 2.36 mm, and alveolar bone thickness below 0.45 mm. 

A key limitation across this domain is that the literature measures different things and often treats them as equivalent. Some studies report plaque or bleeding indices, others report probing depth, others focus on gingival recession, and many orthognathic-periodontal studies still emphasize alveolar imaging changes rather than clinical attachment outcomes. Yet these are not interchangeable endpoints. A patient may show a transient increase in plaque and probing depth during recovery without experiencing long-term recession. Conversely, a patient may complete treatment with acceptable hygiene yet still show a localized, lasting soft-tissue margin change in a high-risk mandibular incisor region. The strongest conclusion, therefore, is not that post-treatment periodontal outcomes are uniformly favorable or uniformly harmful. It is the short-term inflammatory burden that appears more reproducible than the long-term structural periodontal burden. Overall, the evidence supports three main points. First, small short-term increases in plaque accumulation, probing depth, and localized gingival recession can occur after surgery. Second, these changes do not consistently translate into measurable medium-term deterioration in full-mouth periodontal status. Third, long-term instability seems more likely to be localized and phenotype-dependent than generalized, with the mandibular anterior region remaining the site of greatest concern. What is still missing are prospective studies that follow patients from decompensation through surgery, postsurgical finishing, and retention while using standardized measures of recession, clinical attachment level, and site-specific periodontal stability.

Factors associated with periodontal outcomes

Periodontal prognosis in orthodontic-surgical patients is not determined by surgery alone. The principal factors likely to modify periodontal prognosis during combined orthodontic-orthognathic treatment, together with their proposed biologic rationale, relative evidence strength, and clinical implications, are summarized in Table 2. The available evidence suggests that outcomes are shaped by the interaction among baseline anatomy, the direction and extent of decompensation, periodontal phenotype, and the way treatment is executed across phases. He et al., in their systematic review and meta-analysis, identified incisor proclination as a factor influencing gingival recession during presurgical decompensation, but they did not find consistent associations between bone loss and incisor proclination, vertical facial type, or sex across the pooled studies. That finding alone suggests that no single variable explains prognosis adequately [23]. Malocclusion pattern and jaw-specific anatomy are among the most important modifiers. In broader orthodontic multivariate analysis, Luo et al. found that skeletal Class III was strongly associated with mandibular labial dehiscence after treatment, with an odds ratio of 2.368 relative to skeletal Class I [10]. In the same study, skeletal Class II was associated with mandibular lingual fenestration, with an odds ratio of 2.344. These data are not specific to orthognathic cohorts, but they are useful because they mirror what the orthognathic literature repeatedly suggests clinically. Periodontal risk is not distributed evenly across malocclusion types, and the vulnerable surface changes according to the original compensation pattern and the direction of correction. Vertical pattern further modifies this anatomy-driven risk. Lyu et al. showed in adult skeletal Class II patients undergoing presurgical orthodontics that the hyperdivergent group had the thinnest lingual alveolar bone at the apical and midpoint levels before treatment [38]. They also found that root movement during decompensation significantly influenced the morphology of the lingual alveolar bone after treatment. In practical terms, hyperdivergent patients may tolerate less root movement toward the lingual cortical boundary than hypo- or normodivergent patients. This helps explain why similar orthodontic objectives may produce different periodontal consequences in different facial types. Movement magnitude matters, but movement direction and root position appear to be more important. Su et al. studied 60 patients with skeletal Class III high-angle malocclusion and found that presurgical decompensation reduced alveolar bone thickness and vertical height around the mandibular central incisors [39]. The prevalence of labial bone defects increased from 51.7% to 73.3% in the mandibular left central incisor and from 51.7% to 76.7% in the right central incisor. They also reported that when the probability of bone-defect occurrence was set at 50%, the thresholds for change in incisor mandibular plane angle (IMPA) and lower incisor to NB line (L1-NB) were only 5.47° and 5.91°, respectively.

**Table 2:** Modifying factors affecting periodontal prognosis in orthodontic patients undergoing orthognathic surgery CBDT: cone-beam computed tomography

Factor	Biologic rationale	Clinical implication	Supporting references
Thin periodontal phenotype	Reduced soft-tissue thickness and limited resistance to marginal instability may increase susceptibility to recession during decompensation or after surgery, especially when tooth movement approaches cortical limits	Assess phenotype early. Thin-phenotype patients may require closer periodontal monitoring, more conservative mechanics, and selective periodontal consultation or phenotype modification in high-risk sites	Kao et al., 2020 [4]; Kadkhodazadeh et al., 2024 [5]; Jing et al., 2019 [20]; Liu et al., 2024 [14]; Fleming and Andrews, 2024 [40]; Li et al., 2024 [41]
Limited alveolar housing or thin cortical boundaries	A restricted dentoalveolar envelope reduces the biologic tolerance for labiolingual root movement and may predispose to dehiscence, fenestration, or reduced alveolar support	Evaluate alveolar boundaries before extensive decompensation, particularly in the mandibular anterior region. Consider CBCT selectively in high-risk cases, while interpreting radiographic defects together with clinical findings	Kook et al., 2012 [15]; Kim et al., 2009 [16]; Oh et al., 2020 [17]; Yagci et al., 2012 [18]; Patcas et al., 2012 [21]; Alsino et al., 2022 [22]; Lee et al., 2012 [24]; Sun et al., 2015 [25]
Mandibular incisor position	Mandibular incisors are commonly located near thin cortical plates in skeletal malocclusion patients, particularly in compensated Class III cases; decompensation may further challenge these sites	The mandibular anterior region should be considered the principal periodontal risk site during treatment planning and monitoring	Kook et al., 2012 [15]; Kim et al., 2009 [16]; Oh et al., 2020 [17]; He et al., 2024 [23]; Lee et al., 2012 [24]; Liu et al., 2024 [14]; Su et al., 2026 [39]
Magnitude and direction of incisor movement	Movement toward the cortical boundary, rather than movement magnitude alone, appears to be the main determinant of periodontal risk. Root displacement may reduce support on one surface even if another surface appears to improve	Plan decompensation according to root position within the alveolar envelope, not cephalometric objectives alone. Avoid overdecompensation in anatomically constrained patients	He et al., 2024 [23]; Lee et al., 2012 [24]; Sun et al., 2015 [25]; Yao et al., 2020 [26]; Demirsoy et al., 2022 [27]; Fu et al., 2023 [28]; Su et al., 2026 [39]
Vertical facial pattern	Hyperdivergent patients may present with thinner alveolar bone and less favorable support around incisors, making them more vulnerable during decompensation	Vertical pattern should be incorporated into periodontal risk assessment, especially in high-angle Class II and Class III patients	Han et al., 2024 [19]; Yao et al., 2020 [26]; Fu et al., 2023 [28]; Lyu et al., 2023 [38]; Su et al., 2026 [39]
Type of skeletal malocclusion	Different skeletal patterns are associated with different baseline compensation patterns and therefore different periodontal risk surfaces during decompensation	Risk assessment should be individualized according to malocclusion type rather than generalized across all orthognathic patients	Yagci et al., 2012 [18]; Luo et al., 2024 [10]; Kook et al., 2012 [15]; Kim et al., 2009 [16]; Oh et al., 2020 [17]; Fu et al., 2023 [28]; Lyu et al., 2023 [38]
Pre-existing recession or mucogingival deficiency	Existing soft-tissue compromise may reduce the ability of the periodontium to tolerate additional orthodontic or surgical stress	Sites with baseline recession or limited keratinized tissue should be documented before treatment and followed closely throughout decompensation and recovery	Kao et al., 2020 [4]; Renkema et al., 2013 [7]; Mester et al., 2026 [13]; Mota de Paulo et al., 2020 [35]; Fleming and Andrews, 2024 [40]; Liu et al., 2024 [14]
Oral hygiene and plaque control	Postoperative discomfort, edema, splints, and appliances may impair plaque control and contribute to transient inflammation, increased probing depth, or marginal instability	Reinforced hygiene support is essential, especially in the immediate postoperative period and in patients with fixed appliances	Weinspach et al., 2012 [31]; Esen, 2022 [36]; de Leyva et al., 2023 [37]
Treatment duration	Longer treatment may prolong plaque-retentive conditions and extend the duration of periodontal stress, particularly in adult patients	Efficient treatment sequencing and avoidance of unnecessary prolongation may help reduce cumulative periodontal burden	Renkema et al., 2013 [7]; Luo et al., 2024 [10]; de Leyva et al., 2023 [37]
Orthodontic mechanics	Mechanics that produce uncontrolled tipping or excessive root movement toward cortical plates may increase alveolar and soft-tissue risk	Periodontal safety should influence biomechanics selection, especially during presurgical decompensation	He et al., 2024 [23]; Lee et al., 2012 [24]; Sun et al., 2015 [25]; Yao et al., 2020 [26]; Demirsoy et al., 2022 [27]; Fu et al., 2023 [28]; Su et al., 2026 [39]
Transverse correction	Buccal displacement during transverse coordination may challenge facial cortical support in anatomically thin sites, although direct orthognathic-specific evidence remains limited	Expansion and transverse decompensation should be approached cautiously in thin-phenotype or narrow-housing patients	Guo et al., 2026 [6]; Luo et al., 2024 [10]; Fleming and Andrews, 2024 [40]
Segmental osteotomy	Interdental osteotomies may create site-specific periodontal risk through root proximity, interdental bone compromise, and papillary or osseous defects	Segmental procedures require careful case selection, root positioning, and periodontal surveillance around osteotomy sites	Schultes et al., 1998 [32]; Ueki et al., 2006 [33]; Ho et al., 2011 [34]
Nonsegmental orthognathic surgery	Usually contributes more to transient postoperative inflammatory burden than to major independent periodontal breakdown	Monitoring should focus on early healing, hygiene support, and differentiation between temporary inflammatory change and persistent structural damage	Weinspach et al., 2012 [31]; Mota de Paulo et al., 2020 [35]; Esen, 2022 [36]; Mester et al., 2026 [13]
Appliance type during finishing	Appliances influence plaque retention and access for oral hygiene; removable systems may favor better periodontal indices during recovery and finishing	Appliance selection may be relevant in periodontally susceptible patients, particularly during the postsurgical phase	de Leyva et al., 2023 [37]
Age	Older patients may have less periodontal reserve and a higher baseline prevalence of mucogingival defects	Adult orthognathic patients may benefit from more detailed baseline periodontal assessment and longer follow-up	Renkema et al., 2013 [7]; Luo et al., 2024 [10]; Mester et al., 2026 [13]
Adjunctive periodontal/augmentation procedures	Soft-tissue augmentation or corticotomy-assisted augmentation may increase tissue thickness or improve alveolar support in selected high-risk sites	These procedures should not be considered routine, but may be useful in carefully selected thin-phenotype or high-risk mandibular anterior cases	Kao et al., 2020 [4]; Guo et al., 2026 [6]; Ahn et al., 2012 [29]; Ma et al., 2023 [30]; Liu et al., 2024 [14]; Li et al., 2024 [41]

These values suggest that the biologically safe range for mandibular incisor decompensation may be relatively narrow in high-angle Class III patients with thin alveolar housing. However, such thresholds should be interpreted as probabilistic rather than universal. Demirsoy et al. found no significant linear association between the amount of incisor inclination change and defect formation, suggesting that the same angular movement may have different periodontal consequences depending on baseline anatomy [27]. A small movement in an ultra-thin cortical envelope may produce dehiscence or fenestration, whereas a larger movement may be tolerated when alveolar housing and periodontal phenotype are more favorable. Thus, inclination change is clinically relevant, but it should be interpreted together with root position, alveolar thickness, vertical pattern, and soft-tissue phenotype. Periodontal phenotype remains a major biologic determinant. Fleming et al. emphasized that a thin periodontal phenotype is associated with increased recession risk during orthodontic treatment, particularly when treatment involves ambitious arch lengthening by proclination or expansion [40]. They also noted that tooth-specific planning and combined orthodontic-periodontal input become especially important in anatomically susceptible patients. In orthognathic Class III patients, Liu et al. provided more direct clinical support for this concept [14]. They found that mandibular incisors were at greater risk of gingival recession after orthodontic-orthognathic treatment when gingival thickness was below 0.72 mm, alveolar bone height exceeded 2.36 mm, or alveolar bone thickness was below 0.45 mm. These thresholds are not universal rules, but they do show that prognosis is strongly conditioned by baseline phenotype and alveolar dimensions. Phenotype modification may therefore alter prognosis in selected high-risk patients. Li et al. reported a preliminary series of four skeletal Class III patients with a thin periodontal phenotype who underwent soft-tissue augmentation before decompensation [41]. Labial gingival thickness increased by approximately 0.42 mm to 2.00 mm after augmentation, and no gingival recession developed during the subsequent presurgical orthodontic phase. In a larger retrospective cohort, Liu et al. found that in patients without PAOO, gingival thickness decreased by 0.15±0.21 mm, and keratinized gingival width decreased by 0.74±0.91 mm after orthodontic-orthognathic treatment [14]. In the PAOO group, gingival thickness instead increased by 0.32±0.28 mm, and keratinized gingival width increased by 2.09±1.51 mm. The percentage of gingival recession increased by 47.62% in the non-PAOO group and was reported to be 14.77 times that of the PAOO group. These studies do not justify routine augmentation in all surgical patients, but they do suggest that periodontal prognosis is modifiable in selected thin-phenotype cases.

Treatment mechanics and hygiene support also appear to influence prognosis, particularly after surgery. In a randomized trial of 28 patients, de Leyva et al. found better periodontal outcomes with clear aligners than with fixed appliances during postsurgical orthodontic treatment, specifically for bleeding on probing, plaque index, and probing depth, while total treatment duration was similar between groups [37]. This finding should not be overstated, but it indicates that periodontal outcomes are not determined only by skeletal pattern and tooth movement. They are also influenced by appliance-related plaque retention and the patient’s ability to maintain oral hygiene during the vulnerable postsurgical healing period. They are also affected by how easily the patient can maintain hygiene during recovery and finishing. By contrast, direct orthognathic-specific evidence for the independent effects of age, sex, and treatment duration remains limited. Broader orthodontic multivariate work by Luo et al. suggested that older age, extraction, and miniscrew use influenced the prevalence of anterior lingual dehiscence, whereas crowding was associated with maxillary labial dehiscence [10]. These variables are likely relevant to orthognathic cohorts as well, but they have not yet been studied with the same clarity within phase-specific orthodontic-surgical designs. For that reason, they should currently be regarded as plausible modifiers rather than established orthognathic-specific determinants.

Overall, the current evidence supports a layered view of periodontal prognosis. The highest risk appears in patients who combine an unfavorable skeletal pattern with a thin phenotype, limited alveolar housing, and decompensatory root movement toward a cortical boundary. The periodontal outcome then becomes more favorable or less favorable depending on whether treatment mechanics remain biologically contained, whether hygiene can be maintained, and whether adjunctive phenotype modification is used in carefully selected cases. This framework also explains why the literature is mixed: prognosis is not governed by one variable, but by the accumulation of several interacting risks across the full orthodontic-surgical pathway.

Implications for clinical practice

Based on the reviewed evidence, periodontal management in orthodontic-surgical patients can be organized into a three-phase interdisciplinary protocol.

Pre-treatment Risk Stratification

Every patient being considered for combined orthodontic-orthognathic treatment should undergo a dedicated baseline periodontal assessment before bonding. This should include periodontal charting, documentation of gingival recession, probing depth, bleeding on probing, keratinized tissue width, clinical attachment level where indicated, periodontal phenotype, and evaluation of alveolar bone support at the intended sites of tooth movement. Patients with a thin periodontal phenotype, hyperdivergent facial pattern, severe baseline dentoalveolar compensation, limited alveolar housing, pre-existing recession, reduced keratinized tissue, or mandibular incisor roots positioned close to the cortical boundary should be classified as periodontally high risk. In such patients, orthodontic-periodontal consultation before active decompensation is warranted, and adjunctive phenotype-modifying procedures, such as soft-tissue grafting or periodontally accelerated osteogenic orthodontics, may be considered selectively rather than routinely [4-7,10,13,14,17-25,29-31,35-41].

Presurgical Decompensation As the Highest-Risk Window

Presurgical orthodontic decompensation should be regarded as the phase in which periodontal hard-tissue risk is most likely to accumulate, particularly around the mandibular incisors. Treatment objectives should therefore be guided by the biologic limits of the alveolar housing rather than by cephalometric norms alone. Root movement toward thin cortical plates should be planned cautiously, tipping should be controlled, and excessive or non-individualized decompensation should be avoided in patients with restricted alveolar support. In this phase, periodontal safety depends not only on the amount of incisor movement, but also on movement direction, root position, baseline cortical thickness, vertical facial pattern, and soft-tissue phenotype [14,23,26-28,38-41].

Post-surgical Hygiene Support and Stability Monitoring

The early postoperative period, particularly the first six weeks after surgery, should be treated as a vulnerable periodontal interval because edema, discomfort, limited mouth opening, surgical splints, intermaxillary elastics, and fixed appliances may impair plaque control. Reinforced oral-hygiene instruction, professional monitoring, and early identification of localized inflammation, increased probing depth, or marginal tissue change should be part of routine postsurgical care. Long-term periodontal follow-up should continue through finishing and retention, especially in patients who had a thin phenotype, alveolar compromise, gingival margin change, or extensive decompensation during treatment, because mucogingival breakdown may become clinically evident after active orthodontic appliances are removed [7,13,14,31,35-37].

Limitations and future research directions

Several methodological concerns recur across the studies included in this review and must be acknowledged explicitly when interpreting its conclusions. First, the majority of included studies are retrospective and observational, with limited sample sizes. Second, outcome measurement is highly heterogeneous. Some studies rely exclusively on CBCT-derived bone measurements, others on clinical parameters such as probing depth, gingival recession, and clinical attachment level, and still others on plaque and bleeding indices. Third, few studies isolate the contribution of individual treatment phases. Most report outcomes at treatment completion, making it impossible to attribute findings to decompensation, surgery, or postsurgical finishing with confidence. Fourth, most studies focus almost exclusively on skeletal Class III malocclusion and on the mandibular anterior region. Evidence on Class II patients, on the maxillary anterior segment, and on posterior regions is substantially thinner and should not be extrapolated from Class III findings without caution. Fifth, the role of surgery-first approaches, which bypass conventional presurgical orthodontic decompensation, is incompletely addressed in the periodontal literature, and the implications of this increasingly used protocol for alveolar bone and soft-tissue outcomes remain substantially uncharacterized. Because this article was a structured comprehensive narrative review rather than a systematic review, no formal risk-of-bias assessment and no Preferred Reporting Items for Systematic Reviews and Meta-Analyses (PRISMA) flow diagram were performed. The search strategy and eligibility criteria were included to improve breadth, but the aim was thematic clinical synthesis rather than quantitative pooling or certainty-of-evidence grading.

The most pressing methodological need is for prospective longitudinal studies that follow patients from baseline through decompensation, surgery, postsurgical finishing, and a minimum of two years of retention, using standardized measurements of clinical attachment level, gingival recession, probing depth, and alveolar bone at predefined sites and timepoints. The current reliance on retrospective cohorts with single endpoint assessments makes it impossible to attribute periodontal findings to specific treatment phases with confidence, and the resulting ambiguity has substantially limited both the interpretation and the clinical applicability of the available evidence. Prospective designs with pre-specified periodontal outcomes as primary or co-primary endpoints are needed to establish the temporal trajectory of periodontal change and to quantify the relative contribution of decompensation, surgery, and postsurgical finishing to the overall burden.

## Conclusions

Periodontal risk in patients undergoing combined orthodontic-orthognathic treatment is a real, multi-phase, and modifiable phenomenon that current evidence can characterize but not yet fully quantify. The evidence most consistently supports the view that the mandibular anterior region is the zone of greatest vulnerability, that presurgical orthodontic decompensation is the phase during which the greatest hard-tissue changes accumulate, and that long-term periodontal harm, when it occurs, is more likely to be localized and phenotype-dependent than generalized. Baseline periodontal susceptibility in skeletal malocclusion patients is not an artifact of treatment; it often predates the first orthodontic intervention and must be identified and integrated into interdisciplinary planning from the outset. At the same time, this review clearly documents that the evidence base is fragmented, predominantly retrospective, concentrated in Class III cohorts, and limited by inconsistent outcome measurement and follow-up duration.
